# Supplementary material for: The interactome of intact mitochondria by cross-linking mass spectrometry provides evidence for coexisting respiratory supercomplexes
Source: Mol Cell Proteomics. 2017 Dec 8;17(2):216–32. doi: 10.1074/mcp.RA117.000470 (PMC5795388; doi:10.1074/mcp.RA117.000470)
Supplement: Supplemental Data [file supp_RA117.000470_133922_0_supp_23978_fzffwf.zip › spectra_annotation/mito_DR_spectra_annotation/87-1-12-1-4-1.pdf]

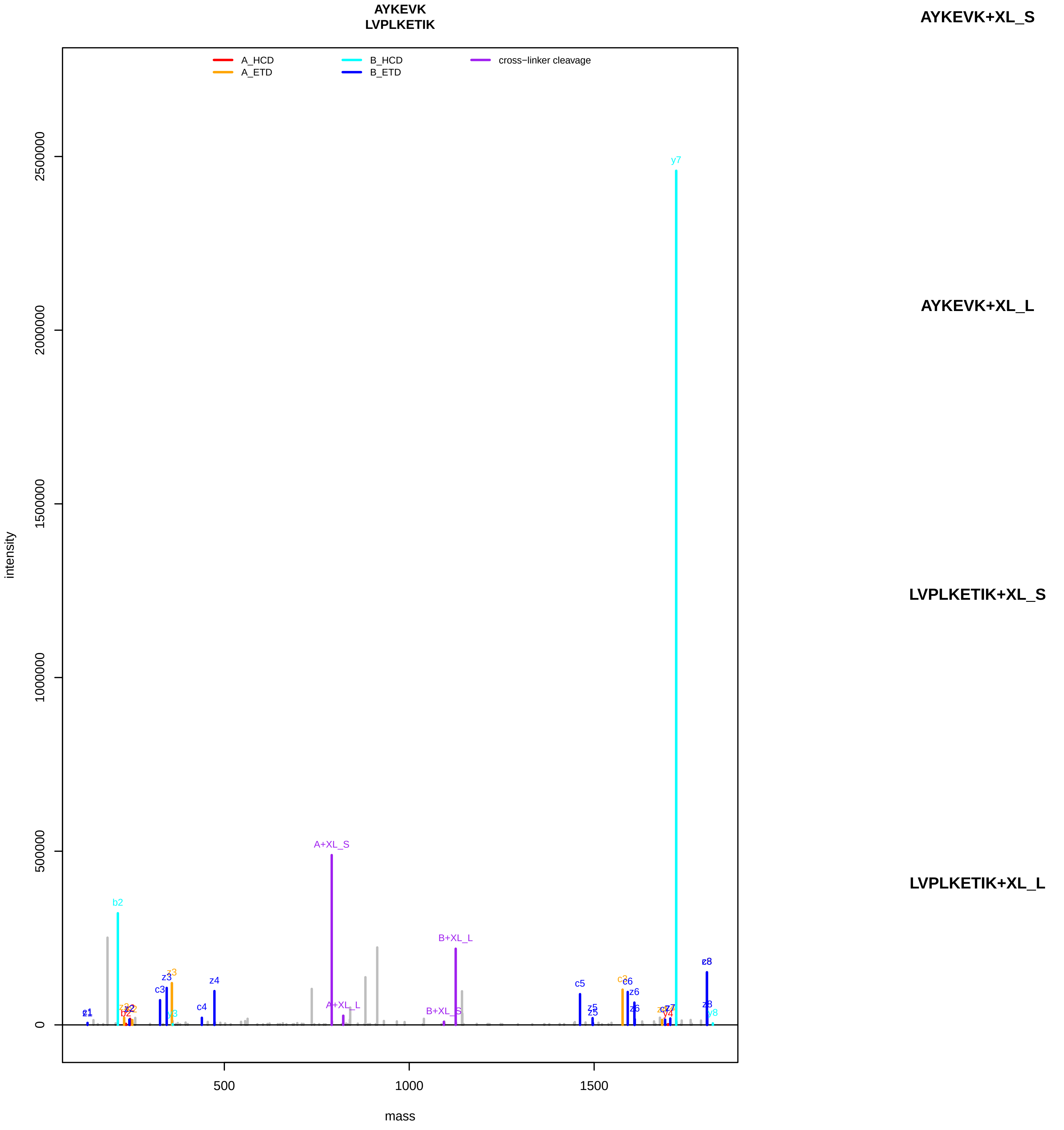
AYKEVK
LVPLKETIK
A_HCD
A_ETD
B_HCD
B_ETD
cross−linker cleavage
intensity
mass
0
500000
1000000
1500000
2000000
2500000
500
1000
1500
y7
b2
A+XL_S
B+XL_L
A+XL_L
B+XL_S
z3
c3
z4
c4
c5
z5
c6
z6
z8
y8
AYKEVK+XL_S
AYKEVK+XL_L
LVPLKETIK+XL_S
LVPLKETIK+XL_L
